# Neonatal Hyperthyroidism Due to TSHR p.Thr632Ile Variant With a Suggestive Familial Pattern

**DOI:** 10.1016/j.aed.2026.06.007

**Published:** 2026-06-17

**Authors:** Ortal Resnick, Owen Chandler, Giovanna Beauchamp

**Affiliations:** 1 Division of Pediatric Endocrinology and Diabetes, University of Alabama at Birmingham, Birmingham, Alabama; 2 Department of Pediatrics, University of Alabama at Birmingham, Birmingham, Alabama

**Keywords:** nonimmune congenital hyperthyroidism, TSH receptor mutation, neonatal thyrotoxicosis, genetic hyperthyroidism, craniosynostosis

## Abstract

**Background/Objective:**

Congenital nonimmune hyperthyroidism due to activating mutations in the thyroid-stimulating hormone receptor (TSHR) is rare and often presents diagnostic and management challenges. We report a case of the *TSHR* p.Thr632Ile variant, with a maternal clinical history highly suggestive of the same underlying disorder, highlighting diagnostic challenges and clinical variability associated with this mutation.

**Case Report:**

A 2-month-old female presented with severe thyrotoxicosis, feeding intolerance, tachycardia, and failure to thrive. Thyroid receptor antibodies were negative, and genetic testing confirmed a heterozygous pathogenic TSHR p.Thr632Ile variant. Her mother had a history of profound infantile hyperthyroidism, early thyroidectomy with regrowth, and recurrence during adolescence requiring radioactive iodine, features consistent with an activating *TSHR* mutation. Both the mother and infant demonstrated neurodevelopmental delays.

**Discussion:**

This report provides detailed phenotyping of a *TSHR* p.Thr632Ile variant, demonstrating persistent severe disease activity, partial resistance to medical therapy, requirement for definitive intervention, and neurodevelopmental impact.

**Conclusion:**

Activating TSHR mutations should be considered in infants with persistent hyperthyroidism. Early genetic diagnosis guides management decisions and genetic counseling.

**Highlights**

• Thyroid-stimulating hormone receptor p.Thr632Ile variant causing severe infantile thyrotoxicosis
• Maternal history suggests a familial phenotype
• Partial resistance requiring high-dose antithyroid therapy
• Thyroid remnant regrowth after subtotal thyroidectomy
• Absence of craniosynostosis despite severe biochemical hyperthyroidism

**Clinical Relevance**

This report expands the phenotypic spectrum of the rare thyroid-stimulating hormone receptor p.Thr632Ile variant by demonstrating severe disease with a suggestive familial pattern, resistance to medical therapy, and thyroid remnant regrowth without craniosynostosis. These findings inform prognosis, surgical planning, and long-term monitoring in affected families.

## Introduction

Congenital hyperthyroidism is rare and may result from transplacental maternal thyroid-stimulating hormone (TSH) receptor antibodies or, less commonly, germline activating mutations in the thyroid-stimulating hormone receptor (TSHR).

Disease pathogenesis results from constitutive activation of the TSHR, leading to ligand-independent stimulation of thyroid follicular cells and unregulated thyroid hormone production.^1^, ^2^, ^3^ The TSHR is a G-protein coupled receptor, with exon 10 encoding the 7 transmembrane helices and the receptor’s activation machinery. The p.Thr632Ile mutation lies within the sixth transmembrane segment of exon 10, a region critical for receptor activation. Activating mutations within this domain have been shown to result in constitutive receptor activation, as demonstrated in early studies of TSHR transmembrane domain variants and chronically stimulate the cyclic adenosine monophosphate signaling pathway, promoting thyroid hormone synthesis and thyroid epithelial proliferation. Functional studies suggest that p.Thr632Ile also activates the phospholipase C/IP_3_ pathway, providing an additional mechanism of intracellular stimulation.^3^, ^4^, ^5^, ^6^, ^7^

Although activating TSHR mutations are well described, only a small number of reports have specifically described the p.Thr632Ile variant, with variability in clinical presentation and severity.^8^, ^9^, ^10^ Here, we describe a case of congenital nonimmune hyperthyroidism with a maternal clinical history highly suggestive of the same underlying disorder, providing additional clinical characterization of this variant, including a severe early-onset presentation and suspected familial involvement.

## Case Presentation

A 2-month-old female presented with persistent diarrhea, tachycardia, and poor weight gain. She was born at 36 weeks following an uncomplicated pregnancy. Her mother had a history of infantile hyperthyroidism requiring thyroidectomy and radioactive iodine ablation and was maintained on levothyroxine.

Newborn screening (NBS) obtained at 1 day of life and again at 1 month of age was both unsatisfactory. At 1 month of age, the infant developed worsening diarrhea and weight loss despite preserved appetite. A third screen at 6 weeks of age reported total thyroxine of 24.2 μg/dL and a TSH <3 μIU/mL, values interpreted as normal by the state program. Thyroid serum testing revealed suppressed TSH with markedly elevated free thyroxine and triiodothyronine levels (Table 1), prompting urgent referral to the emergency department.

**Table 1:** Thyroid Function Tests at Diagnosis

Test	Reference range	Infant (2 mo)	Mother (infancy)
TSH (mIU/L)	1-4.9	<0.01	<0.02
Free T4 (ng/dL)	0.69–1.56	>5.00	—
Total T4 (μg/dL)	4.5–12.0	—	17.8
Total T3 (ng/dL)	85–250	536	556
TRAB (U/L)	<1.5 negative ≥1.5 positive	<0.3	<1.1
TSH binding inhibition index (% inhibition)	<10 negative 11-14 intermediate ≥15 positive	—	14^a^
TSI (% baseline activity)	<140 negative	<89^b^	—

Abbreviations: NBS, newborn screen; T3, triiodothyronine; T4, thyroxine; TRAB, TSH receptor antibody; TSH, thyroid-stimulating hormone; TSI, thyroid-stimulating immunoglobulin.

a Measured at 12 mo of age.

b Measured at 4 mo of age.

At presentation, she demonstrated tachycardia, hypotonia, frontal bossing, and severe undernutrition (weight-for-age Z-score −3.04). Repeat serum testing demonstrated suppressed TSH with markedly elevated free T4 and T3 levels. In the absence of detectable TSH receptor antibodies, these findings were consistent with nonautoimmune hyperthyroidism. Methimazole (0.35 mg/kg/d divided twice daily) and propranolol (2 mg/kg/d administered 3 times daily) were initiated with clinical improvement; however, escalating doses of methimazole (up to 1 mg/kg/d) were required to achieve only partial biochemical control. TSH receptor antibodies (TRAb) were negative in both mother and infant, and thyroid peroxidase antibodies and thyroid-stimulating immunoglobulin were negative in the infant, raising suspicion of a germline activating mutation.

Genetic testing identified a heterozygous variant, c.1895C > T (p.Thr632Ile), likely pathogenic, confirming nonautoimmune congenital hyperthyroidism. No variants of uncertain significance were detected.

Thyroid ultrasound showed diffuse enlargement without suspicious features. Head computed tomography at 8 months demonstrated open sutures without craniosynostosis. At 12 months, she remained clinically stable with improved growth but persistent biochemical hyperthyroidism requiring ongoing medical therapy. Delays in gross motor and communication milestones were noted.

The mother had developed hyperthyroidism in early infancy with persistent diarrhea, tachycardia, and poor weight gain. Laboratory evaluation revealed marked hyperthyroidism (Table 1). She underwent antithyroid medication trials, including propylthiouracil, and methimazole (the latter discontinued due to rash). Propylthiouracil provided transient improvement, but thyroxine repeatedly rebounded. At 14 months of age, she underwent subtotal thyroidectomy and was started on levothyroxine and calcium supplementation. At 3 years of age, she developed recurrent symptoms of hyperthyroidism with laboratory findings confirming hyperthyroidism and cervical lymphadenopathy. Biopsy of an enlarged cervical lymph node demonstrated ectopic thyroid tissue. She underwent surgical excision. She remained stable until 13 years of age, when she experienced recurrent hyperthyroidism and was treated with radioactive iodine ablation. Since then, she has been stable and treated with levothyroxine. She reports lifelong learning difficulties. Aside from the affected mother and infant, there is no family history of hyperthyroidism.

## Discussion

Congenital hyperthyroidism is a rare but clinically significant diagnosis that requires prompt recognition to prevent long-term morbidity. Although most neonatal thyrotoxicosis is transient and caused by maternal TRAb, a small subset results from germline activating mutations in the *TSHR* or *GNAS* gene mutation associated with McCune-Albright syndrome. Hyperthyroidism from McCune-Albright syndrome typically presents with a markedly elevated T3-to-T4 ratio (>20), café-au-lait macules, and fibrous dysplasia, features that were absent in this patient.

*TSHR* mutations cause persistent congenital hyperthyroidism. The current cases provide additional clinical characterization of the p.Thr632Ile variant, which has been reported in a limited number of cases with variable clinical severity.

Early identification and treatment of hyperthyroidism in infancy are critical for preventing adverse neurodevelopmental outcomes. Untreated or poorly controlled neonatal hyperthyroidism can result in craniosynostosis, advanced bone age, failure to thrive, cardiac dysfunction, and persistent cognitive impairment.^11^ Mortality rates of 12% to 20% have been reported in historical cohorts, primarily due to heart failure, tracheal compression, infections, and thrombocytopenia.^12^

Infants born to mothers with Graves’ disease, or with a family history of congenital hyperthyroidism, are at increased risk and should undergo serum thyroid function testing screening in the first and second weeks of life, regardless of NBS results.^13^

In the presented case diagnosis was delayed, serum thyroid function testing was not performed in the early postnatal period as recommended for high-risk infants, and NBS specimens were repeatedly unsatisfactory. In addition, the 6-week screening result was interpreted using neonatal instead of age-appropriate reference ranges. This case emphasizes the importance of adhering to guideline-recommended early serum testing in high-risk infants.

Several features in this infant strongly suggested a nonimmune etiology. Both maternal and infant TRAb levels were negative, and the progressive elevation of thyroid hormone levels in both the mother and infant despite appropriate therapy further supported an intrinsic activating mechanism rather than an immune-mediated process. Maternal antibodies are typically cleared by 20 weeks of age and almost always by 48 weeks.^14^

Only a few reports describe the p.Thr632Ile variant in either familial or sporadic presentations with substantial variability in clinical severity (Table 2).^8^, ^9^, ^10^ Phenotypes range from relatively mild childhood hyperthyroidism with partial improvement after puberty to severe neonatal thyrotoxicosis requiring early definitive therapy.

**Table 2:** Clinical Features of Reported Cases With the *TSHR* p.Thr632Ile Mutation Compared With the Presented Family

Clinical Feature	Chawla^10^ (Child)	Kopp^8^ (Child)	Biebermann^9^ (Child)	Biebermann^9^ (Mother)	Present case: Child	Present case: Mother
Sex	Male	Female	Male	Female	Female	Female
Age at diagnosis	6 mo	After birth	7 mo	12 y	2 mo	Infancy
Presenting symptoms	Craniosynostosis, FTT	Preterm, jitteriness, tachycardia, CHF	Hyperthyroidism	Hyperthyroidism	Diarrhea, tachycardia, weight loss, FTT, hypotonia, frontal bossing	Diarrhea, tachycardia, weight loss, FTT
Craniosynostosis	Yes - squamosal	Yes - sagittal	Yes	No	No	No
TSHR mutation	p.Thr632Ile heterozygous	p.Thr632Ile heterozygous	p.Thr632Ile	p.Thr632Ile	p.Thr632Ile, heterozygous	Not tested
Surgeries	Thyroidectomy at 13 mo	Near-total thyroidectomy at 3 y; excision of remnant at 14 y	Craniosynostosis surgeries at 2.5 & 5 y	None	None	Subtotal thyroidectomy at 1 y, excision of remnant at 3 y, RAI at 13 y
Neurodevelopmental outcome	Developing age appropriately	Cognitive delay	N/A	N/A	Developmental delay	Learning difficulties

Abbreviations: CHF, congestive heart failure; FTT, failure to thrive; mo, months; RAI, radioactive iodine; yr, years.

In this family, hyperthyroidism manifested in both the mother and child during early infancy, with significant weight loss, tachycardia, and feeding intolerance. The mother’s clinical course, marked by persistent hyperthyroidism, resistance to antithyroid medications, and regrowth of thyroid tissue after surgery, has been described in other patients harboring the p.Thr632Ile mutation.^8^

The somatic occurrence of the p.Thr632Ile mutation in toxic thyroid adenomas has been associated with dual activation of the cyclic adenosine monophosphate and phospholipase C signaling pathways. This enhanced intracellular stimulation may contribute to the more aggressive clinical behavior observed in some patients. Biebermann et al published familial case involving this mutation with a few key differences; the age at onset was older, the disease severity diminished after puberty, and definitive therapy such as thyroidectomy or radioactive iodine was not reported.^9^ In contrast, de novo cases reported in infancy have generally been more aggressive.^8^^,^^10^ Although maternal genetic testing is still needed to confirm inheritance, this family’s presentation suggests that the familial p.Thr632Ile variant may manifest with an aggressive and refractory phenotype.

Neurodevelopmental findings parallel earlier reports involving this mutation. The infant exhibited delays in early motor and communication milestones, and the mother described lifelong learning difficulties. Although neurodevelopmental outcomes in these cases are influenced by multiple factors, prolonged periods of uncontrolled thyrotoxicosis are known to impair cognitive development.^11^

Interestingly, despite the severity of the patients’ biochemical hyperthyroidism, the infant did not develop craniosynostosis on computed tomography scan, and the mother likewise never required surgical intervention for craniosynostosis, an abnormality reported in several prior cases of p.Thr632Ile.^8^, ^9^, ^10^

A limitation of this report is the absence of genetic testing in the mother, and therefore familial transmission cannot be definitively confirmed. This report demonstrates that thep.Thr632Ile variant may manifest with an aggressive and refractory phenotype comparable to de novo neonatal cases, expanding previously reported genotype phenotype correlations. This case underscores several essential principles in the evaluation of infants at risk for hyperthyroidism. Serum thyroid testing should be performed in any infant with a family history of congenital hyperthyroidism or maternal Graves’ disease. In infants with persistent or worsening thyrotoxicosis and negative TRAb early consideration of germline *TSHR* mutations is crucial. Timely diagnosis facilitates appropriate treatment, informs decisions regarding definitive therapy, and enables targeted testing and counseling of at-risk relatives. Additional case descriptions may help further delineate the clinical spectrum associated with this variant.

## Ethical Considerations

Written informed consent for publication was obtained from the patient’s parent/legal guardian and from the mother described in this report.

## Disclosure

The authors have no conflicts of interest to disclose.
